# Exploring tonsillar cancer associations in patients with base of tongue cancer: insights from a single-center study

**DOI:** 10.1007/s00405-024-08830-7

**Published:** 2024-07-23

**Authors:** Sebastian Udholm, Nina B. Sannino-Greve, Tejs Ehlers Klug

**Affiliations:** Department of Otorhinolaryngology, Head and Neck Surgery, Aarhus N. University Hospital, Aarhus, DK-8200 Denmark

**Keywords:** Base of tongue, Head and neck cancer, Carcinoma, Malignancy, Secondary cancer, Cancer

## Abstract

**Purpose:**

To explore the prevalence of synchronous and metachronous tonsillar cancer in patients with base of tongue cancer, as well as identifying potential risk factors linked to these secondary malignancies. We aim to answer the following question: Should bilateral tonsillectomy be recommended to patients diagnosed with base of tongue cancer?

**Methods:**

A case-series study was conducted at Aarhus University Hospital, including all patients with histologically confirmed base of tongue squamous cell carcinoma treated between January 2012 and December 2021. Data from electronic patient records, including diagnosis of prior, synchronous or metachronous tonsillar cancer, demographics, and clinical features were analysed. Fisher’s exact test was performed to assess factors associated with synchronous and metachronous tonsillar cancer.

**Results:**

Among 198 patients with base of tongue cancer, 5.6% had a history of tonsillar cancer, either prior to (4.5%), synchronous (0.5%), or metachronous (0.5%) to the base of tongue diagnosis. The prevalence of synchronous or metachronous tonsillar cancer among patients without previous bilateral tonsillectomy was 1.2%. Patients with tonsillar cancer were older, had heavier smoking histories, and exhibited less frequent P16-overexpression.

**Conclusion:**

Our findings deepen understanding of tonsillar cancer in patients with base of tongue cancer. The prevalence of synchronous or metachronous tonsillar cancer was found to be relatively low, suggesting that routine tonsillectomy for all base of tongue cancer patients is not warranted.

## Introduction

The risk of developing a second primary malignancy is increased in patients with head and neck cancer [1, 2]. These malignancies may manifest either synchronous (diagnosed either at the same time as the primary lesion or within six months) or metachronous (more than six months after the primary cancer). Occurrence of secondary primary malignancies constitute major clinical issues as they impact patients prognosis negatively; indeed, these malignancies are one of the leading causes of death in patients with head and neck cancer [3]. In newly diagnosed head and neck cancer patients, the occurrence of synchronous malignancies is estimated to be in the range 1–6%, while metachronous malignancies occur in almost one third of patients [4–7].

As such, it has long been recognised that individuals with head and neck squamous cell carcinoma face an increased susceptibility to developing second primary malignancies. Only few studies have, however, investigated the locations and specific risks associated with synchronous and metachronous second primary malignancies across the spectrum of head and neck subsites, including patients with base of tongue cancer [8–10]. Although base of tongue cancer, a subset of oropharyngeal malignancies, is garnering increasing attention due to its rising incidence and complex clinical nature, the prevalence and characteristics of synchronous and metachronous tonsillar cancer in these patients remain unexplored [11].

It is important to identify the risk profile linked to these secondary malignancies in patients with base of tongue cancer to enhance patient counselling, improve management, and better guidance in the planning of follow-up. Given that bilateral tonsillar cancer is relatively common, we hypothesised that the frequency of tonsillar cancer would also be considerable among patients with base of tongue cancer [12]. We undertook the current study to explore the prevalence of synchronous and metachronous tonsillar cancer in patients with base of tongue cancer to answer the following question: Should bilateral tonsillectomy be recommended to patients diagnosed with base of tongue cancer? In addition, we aimed to characterise patients with base of tongue cancer with or without associated tonsillar cancer.

## Methods

The study was approved by the Danish Data Protection Agency (1-16-02-94-22) and the Danish Safety Authority (1-45-70-42-22). The requirement for informed consent was waived given the nature of the study.

### Study population

This is a case-series, including all patients with histologically confirmed base of tongue squamous cell carcinoma (DC019) treated at the Department of Otorhinolaryngology, Head and Neck Surgery, Aarhus University Hospital. The study period was from January 1st 2012 until December 31st 2021.

### Data sources

We examined the medical records of all patients using the electronic patient journal. A standardized extraction form was made, including age, sex, clinical symptoms, treatment modalities, tobacco history, alcohol consumption, prior, synchronous and metachronous tonsillar cancer, and histological reports.

### Outcomes

Our main outcome was the occurrence of synchronous or metachronous tonsillar cancer (DC09*) in patients with base of tongue squamous cell carcinoma. In addition, we decided to include patients with tonsillar cancer prior to the index cancer in the description of patients with both base of tongue and tonsillar cancer. We defined synchronous cancer as those diagnosed either at the same time as the index-cancer or within six months, metachronous cancer as those diagnosed more than six months after the index-cancer, and previous cancer as those diagnosed prior to the index-cancer. Secondary outcomes were factors potentially associated with prior, synchronous and metachronous tonsillar cancer. These were analysed for trends and associations.

### Statistical analyses

Continuous variables were reported as means or medians and SD. Comparisons of means were performed by unpaired *t*-test. Categorical variables were summarised by percentages or frequencies. Comparisons of categorical baseline characteristics between patient groups were performed by the Fisher’s exact test. The normality of data was assessed using quantile-quantile plots. Statistical significances were defined as a p value < 0.05. All analyses were performed using Stata 15 (StataCorp LP, TX).

## Results

### Patient characteristics

A total of 198 patients with histologically confirmed base of tongue squamous cell carcinoma were included in this study. Patient characteristics are summarized in Table 1. Among cases, the mean age was 62 years (range 41–83) and 140 (71%) patients were males. Ninety (46%) patients were active tobacco smokers at the time of diagnosis. The quantitative volume of smoking (in pack years) was available in 118 (60%) patients. No significant differences in patient characteristics were observed between males and females. 18% (36/198) of patients had a history of bilateral tonsillectomy.

**Table 1:** Characteristics of patients with base of tongue squamous cell carcinoma

	Cases *n* = 198
Males, n (%)	140 (71%)
Age, mean years (SD)	62.1 (8.3)
P16-overexpression, n (%) ^1^	126/188 (67%)
Tonsillar cancer, n (%)	11 (5.6%)
Prior to BOT, n	9
Synchronous, n	1
Metachronous, after BOT, n	1
Tobacco smoking	
Current, n (%)	90 (46%)
Previous, n (%)	57 (29%)
Never, n (%)	51 (26%)
Pack years, mean^2^ (SD)	40 (20)
Alcohol abuse^3^, n (%)	60 (30%)
Follow up, months, median (range)	37.6 (0.2–104)

BOT = Base of tongue

^1^ P16 status was unavailable in 10 (5.1%) patient cases

^2^ For previous and current smokers

^3^ Defined as > 14 units per week for > 1 year

The median follow-up time was 3.1 years (range: 0.2–8.7). P16-overexpression was noted in the histology reports in 126 (64%) patients, while 62 (31%) tumours were p16-negative, and P16-status was unknown in the remaining 10 (5%) cases. P16 overexpression was significantly more frequent among non-smoking patients (49/51, 96%) compared to former or current smokers (40/77, 52%) (*p* < 0.001).

### Prevalence of previous, synchronous and metachronous tonsillar cancer

Tonsillar cancer was diagnosed in 11 (5.6%) cases, either prior to base of tongue diagnosis (*n* = 9, 4.5%), synchronous (*n* = 1, 0.5%) or metachronous (*n* = 1, 0.5%) (Table 2). The prevalence of synchronous or metachronous tonsillar cancer among base of tongue cancer patients without previous bilateral tonsillectomy (*n* = 162) was 1.2%. In the nine patients with tonsillar cancer prior to base of tongue cancer, the median time between the two cancers was 8.7 years (range: 4.5–17.6).

**Table 2:** Comparison of patients diagnosed with base of tongue cancer with and without associated tonsillar cancer

	Tonsillar cancer		
	Yes	No	p-value
Tonsillar cancer, n (%)	11 (6%)	187 (94%)	
Prior to BOT cancer, n (%)	9 (4.5%)		
Synchronous, n (%)	1 (0.5%)		
Metachronous, n (%)	1 (0.5%)		
Males, n (%)	6 (55%)	134 (72%)	0.30
Age, mean years (SD)	69.0 (5.0)	61.7 (8.3)	0.005
P16-overexpression, n (%)	3 (27%)	123 (69%)^1^	0.006
Tobacco smoking, n (%)			
Current	5 (46%)	85 (45%)	1.0
Previous	5 (46%)	52 (28%)	0.30
Never	1 (9%)	50 (27%)	0.29
Pack years, mean^2^ (SD)	53 (15)	38 (20)	0.021
Excessive alcohol consumption^3^, n (%)	5 (45%)	55 (29%)	0.31
Follow up, median months (range)	21 (0.2–63)	39 (0.5–104)	0.049

BOT = Base of tongue

^1^ P16 status was unavailable in 10 (5.05%) patient cases

^2^ For previous and current smokers

^3^ Defined as > 14 units per week for > 1 year

### Factors associated with previous, synchronous and metachronous tonsillar cancer

Compared to patients without associated tonsillar cancer, patients diagnosed with previous, metachronous or synchronous tonsillar cancer were older (mean 69.0 vs. 61.7 years, *p* = 0.005), those that were smokers (current or previous) had a heavier smoking load (53 vs. 38 pack years, *p* = 0.021), and the tumour exhibited less frequently P16-overexpression (27% vs. 69%, *p* = 0.006) (Table 2). The patient with synchronous tonsillar cancer was a 59-years old former smoker (37 pack years) and a P16-positiv tumour, whereas the patient with metachronous cancer (36 months after the index cancer) was 70-years old and actively smoker (45 pack years) and a P16-negative tumour.

### Treatment modality

89% (177/198) of patients received curatively intended treatment, either chemo-radiotherapy (*n* = 96, 49%), radiotherapy (*n* = 62, 31%), or surgical resection (*n* = 24, 12%). Palliative care was chosen in 21 (11%) cases. The one BOT cancer patient with synchronous tonsillar cancer received curatively intended radiotherapy whereas the patient with metachronous tonsillar cancer received curatively intended chemo-radiotherapy. Among patients with a tonsillar cancer prior to their BOT cancer, five received chemo-radiotherapy and four received radiotherapy as treatment for their primary tonsillar cancer (all curatively intended).

## Discussion

To our knowledge, the current study is the first to estimate the prevalence of synchronous and metachronous tonsillar cancer among patients with base of tongue cancer. We examined the prevalence of synchronous and metachronous tonsillar cancer among patients with base of tongue cancer covering a follow-up period of up to 9 years. In total, 1.0% (2/198) of patients with base of tongue cancer were diagnosed with synchronous and metachronous tonsillar cancer. Deducting the 36 patients with previous bilaterally tonsillectomy, 1.2% (2/162) of patients at risk for developing secondary tonsillar cancer did so in the follow up period. With an aim to describe the full association between base of tongue and tonsillar cancer, and thus including the nine patients with tonsillar cancer prior to base of tongue cancer, 5.6% (11/198) of patients with base of tongue cancer were diagnosed with tonsillar cancer. In this latter cohort, patients were older age, those that were smokers had increased tobacco consumption, and P16 positivity were less prevalent compared to base of tongue cancer patients without associated tonsillar cancer.

The occurrence of simultaneous second primary cancers in individuals with head and neck cancer has previously been found in the range 1–6% [5, 13–15]. Our data demonstrate that approximately 1 in 20 patients with base of tongue cancer have had or developed associated tonsillar malignancy, with only two of the cases occurring after the index cancer (mean follow up 37.6 months). The vast majority of previous studies investigating the prevalence of synchronous cancer in patients with oropharyngeal cancer have focused on patients with primary tonsillar cancer or they did not differentiate between subsites [16]. Several studies suggests that patients who develop second primary malignancy within the oropharynx are more prone to having initial tonsil cancer and developing their second primary in the contralateral tonsil [9, 12, 17]. Only few researchers have addressed the occurrence of second primary among patients with base of tongue cancer, and they focused on HPV-positive cases treated with transoral robotic surgery [9, 18]. Though not estimating the prevalence, Strober et al. recently concluded, that the tonsils are the most common site for secondary malignancies in patients with HPV-positive base of tongue cancer [9]. Stepan et al. found that base of tongue cancer was the most frequent index cancer among patients, who developed secondary oropharyngeal malignancy, demonstrating that 1.2% (5/412) of patients with oropharyngeal cancer developed secondary tonsillar cancer. Unfortunately, a prevalence and patient characteristics of synchronous and metachronous tonsillar cancer in patients with base of tongue cancer was not provided [18]. 

In our cohort of 162 patients, who were not bilaterally tonsillectomized at time of base of tongue diagnosis, only two patients (1.2%) developed tonsillar cancer, suggesting that the risk of a secondary tonsillar cancer in bases of tongue cancer patients is low. This prevalence is likely to be somewhat underestimated with a median follow-up time of 3.1 years, with regards to the fact that the mean time from tonsillar cancer to secondary base of tongue cancer diagnosis was 8.7 years in our cohort.

Overall, individuals with head and neck squamous cell carcinoma face significantly increased risk of developing second primary malignancy. This increased risk is associated to both patient and tumor characteristics. In our study, older age, tobacco load, and P16-negative tumor was significantly associated with dual (both base of tongue and tonsillar) malignancy. These findings are in line with previous studies finding lower risk of simultaneous second primary malignancy among non-smoking patients with HPV-positive oropharyngeal cancer [10, 19]. Indeed, recent studies suggest that patients with HPV-positive oropharyngeal cancer exhibit the lowest risk of simultaneous second primary malignancies among all head and neck cancer patients [19–21]. Although all patients with dual malignancies in the present study received either chemo-radiotherapy or radiotherapy, our study is underpowered to conclude on the effects of different treatment modalities concerning the occurrence of secondary primaries. Even with higher numbers, the results would likely be biased because cases with advanced disease are not suitable for surgical treatment.

### Limitations

While informative, our study has limitations to consider. Firstly, reliance solely on medical records for data collection may have resulted in incomplete or inaccurate information due to inconsistent documentation. Secondly, distinguishing second primary from recurrence is challenging. An experienced consultant reviewed all relevant records and scans to differentiate between second primaries from recurrences. With only one case of metachronous tonsillar cancer diagnosed 3 years after base of tongue cancer and a mean time of 8.7 years (and a minimum of 3.5 years) between prior tonsillar cancer and base of tongue cancer, we believe that the risk of falsely categorizing patients as having second primary is very little. Lastly, statistical power may be insufficient for detecting smaller associations, and our study period may not have allowed adequate follow-up for certain outcomes.

## Conclusion

Our study found that 1.2% of patients with base of tongue cancer without previous tonsillectomy developed synchronous or metachronous tonsillar cancer, rising to 5.6% when including those with prior tonsillar cancer. Patients with both cancers (regardless of temporal relation) were older, had heavier smoking histories, and showed less frequent P16-overexpression. Based on our findings, we do not recommend tonsillectomy for all patients diagnosed with a base of tongue cancer.

## Data Availability

The data that support the findings of this study are available from the corresponding author upon reasonable request.
